# Identification of biomarkers of response to preoperative talazoparib monotherapy in treatment naïve g*BRCA*+ breast cancers

**DOI:** 10.1038/s41523-022-00427-9

**Published:** 2022-05-10

**Authors:** Xuan Liu, Zhongqi Ge, Fei Yang, Alejandro Contreras, Sanghoon Lee, Jason B. White, Yiling Lu, Marilyne Labrie, Banu K. Arun, Stacy L. Moulder, Gordon B. Mills, Helen Piwnica-Worms, Jennifer K. Litton, Jeffrey T. Chang

**Affiliations:** 1 Department of Integrative Biology and Pharmacology, University of Texas Health Science Center at Houston, Houston, TX USA; 2 Department of Immunology, The University of Texas MD Anderson Cancer Center, Houston, TX USA; 3 Department of Translational Molecular Pathology, The University of Texas MD Anderson Cancer Center, Houston, TX USA; 4 Department of Anatomical Pathology, The University of Texas MD Anderson Cancer Center, Houston, TX USA; 5 Department of Systems Biology, The University of Texas MD Anderson Cancer Center, Houston, TX USA; 6 Department of Breast Medical Oncology, The University of Texas MD Anderson Cancer Center, Houston, TX USA; 7 Department of Genome Medicine, The University of Texas MD Anderson Cancer Center, Houston, TX USA; 8 Department of Cell, Developmental, and Cancer Biology, Knight Cancer Institute, Oregon Health and Science University, Portland, OR USA; 9 Department of Experimental Radiation Oncology, The University of Texas MD Anderson Cancer Center, Houston, TX USA; 10 Department of Bioinformatics and Computational Biology, The University of Texas MD Anderson Cancer Center, Houston, TX USA

**Keywords:** Breast cancer, Tumour biomarkers

## Abstract

Germline mutations in *BRCA1* or *BRCA2* exist in ~2–7% of breast cancer patients, which has led to the approval of PARP inhibitors in the advanced setting. We have previously reported a phase II neoadjuvant trial of single agent talazoparib for patients with germline *BRCA* pathogenic variants with a pathologic complete response (pCR) rate of 53%. As nearly half of the patients treated did not have pCR, better strategies are needed to overcome treatment resistance. To this end, we conducted multi-omic analysis of 13 treatment naïve breast cancer tumors from patients that went on to receive single-agent neoadjuvant talazoparib. We looked for biomarkers that were predictive of response (assessed by residual cancer burden) after 6 months of therapy. We found that all resistant tumors exhibited either the loss of SHLD2, expression of a hypoxia signature, or expression of a stem cell signature. These results indicate that the deep analysis of pre-treatment tumors can identify biomarkers that are predictive of response to talazoparib and potentially other PARP inhibitors, and provides a framework that will allow for better selection of patients for treatment, as well as a roadmap for the development of novel combination therapies to prevent emergence of resistance.

## Introduction

Pathogenic germline variants in *BRCA*1 and *BRCA*2 (g*BRCA*+) confer an elevated risk (57% and 49%, respectively) of developing breast cancer^1^ and are found across breast cancer subtypes^2^. Together, approximately 1.6–7% of all breast cancers have hereditary aberrations in *BRCA*^3,4^, although up to 39% are predicted to be deficient in the homologous recombination (HR) DNA damage repair pathway where *BRCA* are key mediators^5^. PolyADP ribose polymerase (PARP) inhibitors (PARPi) were found to be synthetic lethal in cancers with aberrations in HR mediated by *BRCA*^6^ and multiple PARPi have now been approved for the treatment of several cancer subtypes, including g*BRCA*+ breast cancers^7^.

In g*BRCA*+ breast cancers, the randomized OlympiAD^8^ and EMBRACA^9^ trials both demonstrated that single agent PARPi (olaparib and talazoparib, respectively) increased response rates and progression-free survival compared to physician’s choice of chemotherapy, with substantial improvements in quality of life^10^. While objective response rates were approximately 60% in both trials, resistance eventually developed in most patients and neither trial has shown an improvement in overall survival^11,12^. Recently, PARPi have shown promise as single agent therapy in the neoadjuvant setting achieving a pathologic complete response (pCR) rate of 53%^13^, which is similar to that historically seen with anthracycline/taxane-based chemotherapy. Notably, nearly half of the g*BRCA*+ breast cancers treated with neoadjuvant PARPi did not result in pCR; thus, primary resistance to PARP inhibition remains a substantial problem.

Several PARPi resistance mechanisms have been identified in vitro and in various mouse models. Both olaparib^14^ and talazoparib^15^ are substrates of the ABCB1 multidrug efflux channel^16^, which functions to decrease intracellular concentrations of the drug. PARPi resistance can also be mediated by reinstitution of HR through restoration of *BRCA* function by reversion^17,18^ or hypomorphic *BRCA* mutations^19^, and promotion of homologous recombination through inhibition of the shieldin complex^20,21^, as well as by stabilization^22^ or repriming^23^ of replication forks. The shieldin complex determines the balance between low fidelity non-homologous end joining (NHEJ) that can result in accumulation of further mutations and eventual cell death and HR that is compromised but not completely abrogated by *BRCA* mutations.

Although a range of resistance mechanisms has been demonstrated in models, only a limited number have been linked to resistance to PARPi in the clinical setting. Most commonly seen are secondary *BRCA* mutations that restore the HR repair compromised by g*BRCA* mutations. These mutations have been reported in breast and ovarian cancer tumors^24–26^, and also in circulating cell-free DNA in PARPi or platinum resistant prostate^27^, ovarian^28–31^, and breast^30^ cancers. While reversion of *BRCA* is the most frequently documented mechanism, other mutations that could potentially restore HR have been sporadically reported in clinical samples such as *RAD51C* and *RAD51D* mutations in ovarian cancer^32^, or loss of *TP53BP1* and amplification of *MRE11A* in breast cancer^26^. Reversion of *PARP* trapping may also occur; a *PARP1* mutation that can prevent trapping was seen in a resistant ovarian cancer patient^33^.

Notably, most clinical mechanisms of disease resistance have been studied in more heavily pre-treated patients with disease recurrence or metastasis at the time of PARPi therapy. To identify biomarkers of response and to elucidate mechanisms underlying response and resistance for PARPi in treatment naïve g*BRCA*+ breast cancers, we performed multi-omic (RNA-Seq and whole-exome sequencing (WES)) analysis on 13 treatment-naïve g*BRCA*+ tumors from a neoadjuvant clinical trial of the single-agent PARPi talazoparib^13^. Response, determined by residual cancer burden (RCB)^34^, was assessed after six months of treatment. Our results identified possible resistance mechanisms and suggest that biomarkers present in pre-treatment biopsies have the potential to enrich for disease response to single agent talazoparib therapy. Our data may also inform targeted therapy strategies for combination regimens.

## Results

### Genomic profiling of treatment naïve g*BRCA*+ tumors from patients treated with single agent PARPi

As part of an IRB approved protocol, pretreatment core needle biopsies were collected from 20 treatment-naïve patients with BRCA-associated breast cancers who consented to receive neoadjuvant talazoparib monotherapy on clinical trial (Table 1). We excluded one patient who opted to also receive chemotherapy prior to surgery and one patient due to poor RNA data quality, leaving 18 samples in all.

**Table 1:** Description of samples.

Sample	Germline	ER Status	TP53	Response	Subtype	Mutations	Purity	Ploidy
P15	BRCA1	TNBC	G266E	RCB-III	IM	0.67	23%	1.5
P16	BRCA1	ER 5% weak, PR−	E258G	RCB-I		1.17	50%	1.8
P17	BRCA1	TNBC	C141Y	pCR	IM	0.73	48%	1.8
P18	BRCA1	TNBC	R175H	RCB-III	BL2	2.44	48%	3.1
P21	BRCA2	TNBC	17:7,579,310	pCR	M	5.22	81%	3.4
P23	BRCA1	TNBC	R175H	RCB-II	BL2	3.71	34%	1.8
P24	BRCA1	TNBC	L265P	pCR	BL1	3.74	50%	3.1
P25	BRCA1	ER+, PR+, HER2−	wt	RCB-II		2.54	63%	2.0
P26	BRCA1	TNBC	Y220C	pCR	MSL	4.11	43%	1.8
P30	BRCA2	ER+, PR+, HER2−	wt	pCR		2.14	23%	2.0
P31	BRCA1	TNBC	R175H	RCB-II	BL1	3.66	40%	2.2
P34	BRCA1	TNBC	V216M	RCB-II	BL1	1.71	21%	1.7
P36	BRCA2	ER+, PR+, HER2−	wt	RCB-I		2.39	27%	2.3
P19	BRCA1	TNBC		pCR		1.09	0%	2.0
P27	BRCA1	TNBC		RCB-II		0.78	0%	2.0
P29	BRCA1	TNBC		pCR		0.97	0%	2.0
P32	BRCA2	TNBC		pCR		0.98	0%	2.0
P35	BRCA1	ER+, PR−, HER2−		pCR		0.68	0%	2.0

Each sample contains a germline *BRCA*1 or *BRCA*2 mutation (*Germline* column). The *ER Status* column includes the expression of ER, PR, or HER2 based on immunohistochemistry. TNBC (for triple-negative breast cancer) indicates that the patient was negative in all three receptors. Somatic *TP53* mutations were identified by whole-exome sequencing. *wt* indicates no mutation identified. The *Response* column delineates whether the patient was responsive to treatment at surgery, assessed by RCB status. For the TNBC tumors, the TNBC *Subtype* is shown. BL1 is basal-like 1, BL2 is basal-like 2, M is mesenchymal, MSL is mesenchymal stem-like, and IM is immunomodulatory. *Mutations* quantifies the number of non-synomous coding mutations per megabase. *Purity* contains the percent of tumor cells found in the sample, as estimated from the copy number profiles of the exome sequencing. The *Ploidy* of the tumor is also estimated from the exome sequencing.

We then profiled the tumors using whole-exome sequencing (WES) and RNA-sequencing. Copy number analysis with FACETS^35^ revealed a large variation of tumor cellularity. The copy number profiles of five of the 18 tumors were not called, indicating little evidence of copy number changes (Supplementary Fig. S1). An ABSOLUTE^36^ analysis substantiated these results, calling four of these five tumors *non-aneuploid*. The fifth produced only an implausible prediction of a tumor with 100% purity and ploidy = 1, with little evidence of copy number change (Supplementary Fig. S2). Further, these five tumors also had a significantly lower mutation burden (p = 0.01) and no detectable somatic mutations in *TP53*, which is almost universally mutated in g*BRCA*+ breast cancers^37^ (Supplementary Fig. S3). The H&E (hematoxylin and eosin) stained tissue slides from these five tumors confirmed a deficiency of cancer cells in two of them (P32, P35). The remaining three had adequate cancer cell purity (50–70%). The discrepancy between the histology and genomics may be due to spatial heterogeneity within the tumor, and we opted to discard these samples due to uncertainty in the quality of the genomic profiles, leaving 13 tumors for analysis.

This cohort (*n* = 13) included 10 patients with deleterious g*BRCA*1 mutations and three with deleterious g*BRCA*2 mutations. Nine of the 13 (69%) g*BRCA* mutant tumors were triple negative breast cancers (TNBC). 67% (2 out of 3) of the g*BRCA*2 mutant tumors were ER+, while only 20% (2 out of 10) g*BRCA*1 were ER+, a frequency consistent with previous reports^38^. RCB was assessed after patients completed six months of neoadjuvant talazoparib, and we defined response as pCR (38% of tumors) and RCB-I (15%), and resistance as RCB-II (31%) and RCB-III (15%) (Table 1).

**Fig. 1:**
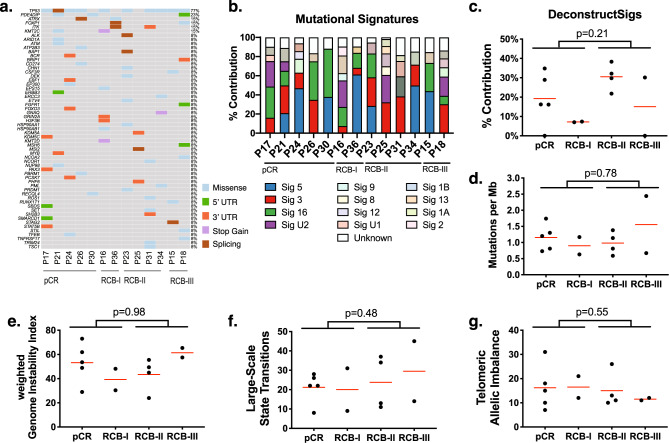
Genomic profile of data set. **a** This oncoplot shows the somatic mutations in cancer-associated genes from the COSMIC database found in this cohort. Each row represents a mutated gene and each column represents a patient. The patients are grouped by RCB. The rectangles are colored according to mutation status. **b** This plot shows the predicted contribution of each mutation signature (*y*-axis), as predicted by DeconstructSigs, to the overall mutational processes in the tumors (columns). The signatures are distinguished by color. **c** The percent contribution of signature 3 (indicative of homologous recombination deficiency) as predicted by DeconstructSigs, are shown on the *y*-axis for each of the tumors. The % contribution from signature 3 is separated according to the response of each tumor. The red line indicates mean contribution, and the *p*-values are calculated using an unpaired two-sided Student’s *t* test comparing pCR and RCB-I against RCB-II and RCB-III. **d** The tumor mutation burden (*y*-axis) is shown for each of the tumors, separated by response. The red lines indicate average mutations. The *p*-values are calculated as in **c**. **e** The weighted genome instability index (*y*-axis) is shown for each tumor, separated by response. The mean indexes are shown by red lines. The *p*-values are calculated as in **c**. **f** The number of large-scale state transitions (*y*-axis) is shown for each tumor, separated by response. The red lines indicate means. The *p*-values are calculated as in **c**. **g** The number of telomeres with imbalanced alleles (*y*-axis) is shown for each tumor, separated by response. The red lines indicate means. The *p*-values are calculated as in **c**.

We called single nucleotide mutations from the WES data and identified 859 non-synonymous somatic mutations with a median of 2.44 mutations per Mb. *TP53* mutations were the most common mutations identified and were present in 10 out of 13 tumors (77%), which is comparable to prior studies showing frequent *TP53* mutations in *BRCA* mutant breast cancers (Fig. 1a)^39^. Nine of the mutations targeted residues in the core DNA-binding domain^40^, while one was predicted to affect splicing. Three tumors harbored R175H hotspot mutations^41^, and three others (G266E, L265P, variant 17:7,579,310) were previously documented in the COSMIC database^42^. All coding region mutations were predicted to be deleterious by both Polyphen2^43^ and SIFT^44^. No other known cancer driver genes were found to be recurrently mutated. However, there were individual cases with mutations in genes or pathways previously associated with PARPi response such as ARID1a, ATM, BAP1, and TSC1.

In addition to driver genes, mutations can also reveal the mutagenic processes that promote the acquisition of mutations throughout the progression of the tumor^45^. *BRCA* mutant breast cancers have defects in double strand break repair that result in mutational patterns characterized as *signature 3*, which has previously been found to be a marker for *BRCA*ness and HR deficiency^46,47^. We confirmed that this signature is seen in this cohort of g*BRCA*+ tumors using SigMA, a tool that was optimized to detect Signature 3, which predicted that this signature contributed 74–85% of the mutational patterns across these tumors^48^ (Fig. S4). However, we found no correlation between this signature and sensitivity to talazoparib (*p* = 0.61), when sensitivity was defined as pCR or RCB-I, and resistance as RCB-II or RCB-III, consistent with the endpoint of the clinical trial that generated these samples^13^; or when comparing pCR and non-PCR tumors (*p* = 0.58). In subsequent analyses, we will primarily defined sensitivity according to the definition from the trial, but also report results from the pCR and non-PCR comparison in the Supplementary Information.

To determine if other processes contribute to the mutations, we repeated the analysis with DeconstructSigs^49^, which can score the contribution of a range of signatures. From this analysis, we found that the mutations across all tumors predominantly reflect signature 3, confirming the SigMA prediction, as well as signatures 5, 16, and U2 (Fig. 1b). The etiology of signatures 5 and 16 is not known, and signature U2 is suspected to be a sequencing artifact^45^. The predictions of signature 3 from the two algorithms were highly correlated (*p* = 0.0004, Supplementary Fig. S4), although the absolute percentages from DeconstructSigs were lower, likely due to contributions from other signatures and artifacts. Accordingly, the predicted contributions from DeconstructSigs also failed to correlate with response (Fig. 1c, Supplementary Fig. S5). It is possible that this signature is best suited for differentiating tumors with and without HR deficiency, and is not sensitive enough to distinguish more subtle degrees of HR competency seen in a cohort consisting entirely of g*BRCA*+ breast cancers.

We next associated tumor mutation burden with response, due to the potential for neoantigens in *BRCA* + breast cancers to contribute to an immune response^50^. However, we found no association between response to PARPi with either tumor mutation burden (Fig. 1d, Supplementary Fig. S6) or with the weighted genome instability index^51^, a measure of genome instability based on copy number aberrations (Fig. 1e). Similarly, we saw no associations between response and other surrogate measures of HRD including large-scale structural chromosomal aberrations, quantified as a large-scale state transition score^52,53^ (Fig. 1f, Supplementary Fig. S7); or telomeric allelic imbalance, asymmetrical copy numbers of the major and minor alleles extending to the telomere, which was predicted to result from failures in double stranded break repair^54^ (Fig. 1g, Supplementary Fig. S8). Taken together, we observed no correlation between measures of HR deficiency and response to single agent talazoparib in this *gBRCA*+ cohort.

### Loss of shieldin complex member SHLD2 is associated with resistance

**Fig. 2:**
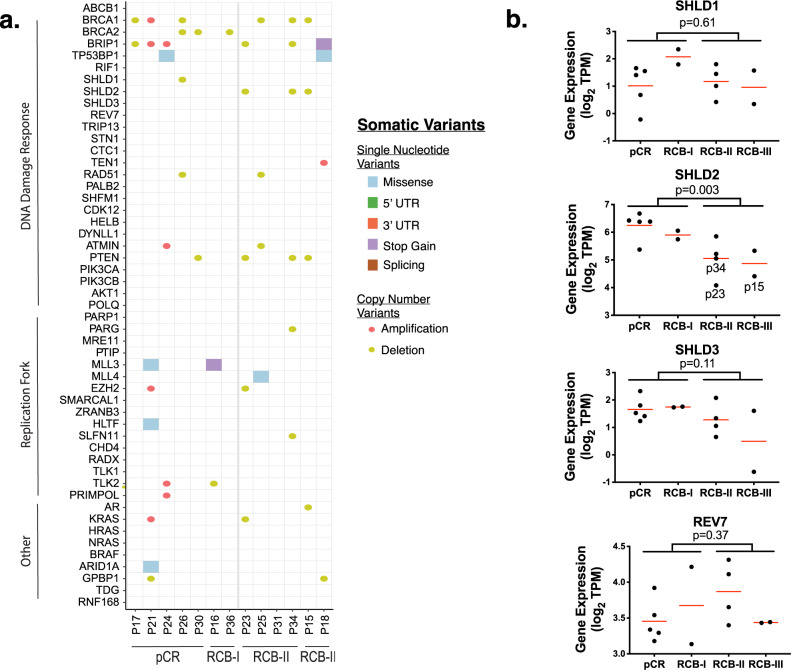
Known resistance genes associated with resistance. **a** This oncoplot shows the mutations seen across a curated list of genes linked with resistance to PARPi. **b** The gene expression of members of the shieldin complex are shown on the *Y*-axis as log_2_ TPM. Individual patients (dots) are grouped according to RCB response. Mean expressions are shown by red lines. The *p*-values are calculated from unpaired two-sided Student’s *t* tests. In the SHLD2 plot, patients predicted to have copy number loss of the gene are labeled.

We next searched for evidence of genomic or transcriptomic changes in 51 genes previously associated with PARPi resistance in pre-clinical models (Table 2). We started by identifying non-synonymous somatic single nucleotide mutations from the WES data (Fig. 2a). We also estimated copy numbers from this data using FACETS^35^ and plotted amplifications or deletions that corresponded with a two-fold deviation from the average expression in responsive tumors. We found no significant structural rearrangements using Manta^55^, although it is highly likely that rearrangements were not well captured in the WES data. In addition, alternative splicing changes can also lead to *BRCA* reversions^56^. However, the RNA-Seq data did not reveal any BRCA splicing events.

**Table 2:** Mechanisms of resistance.

	Gene	Mechanism	Resistance	Reference
	ABCB1	Drug efflux	Gain	Multiple ABCB1 transcriptional fusions in drug resistant high-grade serous ovarian and breast cancer
**DNA** **Damage** **Response**	BRCA1	Promotes DNA end resection.	Gain	Secondary BRCA1 mutations in BRCA1-mutated ovarian carcinomas with platinum resistance
	BRCA2	Promotes homologous recombination.	Gain	Secondary mutations as a mechanism of cisplatin resistance in BRCA2-mutated cancers
	BRIP1	Binds BRCA1.	Gain	Inactivation of the Tumor Suppressor BRIP1 Gene Confers Increased Susceptibility to Platinum Antineoplastic Agents and Augments the Synergistic Response to PARP Inhibition in Ovarian Epithelial Cells
	TP53BP1	Inhibits DNA end resection.	Loss	53BP1 inhibits homologous recombination in Brca1-deficient cells by blocking resection of DNA breaks
	RIF1	Inhibits DNA end resection.	Loss	53BP1 regulates DSB repair using Rif1 to control 5′ end resection
	SHLD1 (C20orf196)	Inhibits DNA end resection. Shieldin complex.	Loss	DNA repair network analysis reveals Shieldin as a key regulator of NHEJ and PARP inhibitor sensitivity
	SHLD2 (FAM35A)	Inhibits DNA end resection. Shieldin complex.	Loss	DNA repair network analysis reveals Shieldin as a key regulator of NHEJ and PARP inhibitor sensitivity
	SHLD3	Inhibits DNA end resection. Shieldin complex.	Loss	DNA repair network analysis reveals Shieldin as a key regulator of NHEJ and PARP inhibitor sensitivity
	REV7/MAD2L2	Inhibits DNA end resection. Shieldin complex.	Loss	MAD2L2 controls DNA repair at telomeres and DNA breaks by inhibiting 5′ end resection
	TRIP13	Promotes dissociation of REV7-Shieldin complex.	Gain	TRIP13 regulates DNA repair pathway choice through REV7 conformational change
	STN1	Inhibits DNA end resection. CST Complex.	Loss	The CST complex mediates end protection at double-strand breaks and promotes PARP inhibitor sensitivity in BRCA1-deficient cells
	CTC1	Inhibits DNA end resection. CST Complex.	Loss	The CST complex mediates end protection at double-strand breaks and promotes PARP inhibitor sensitivity in BRCA1-deficient cells
	TEN1	Inhibits DNA end resection. CST Complex.	Loss	The CST complex mediates end protection at double-strand breaks and promotes PARP inhibitor sensitivity in BRCA1-deficient cells
	RAD51	Stimulates strand invasion. Stabilizes replication forks.	Gain	Secondary somatic mutations restoring RAD51C and RAD51D associated with acquired resistance to the PARP inhibitor rucaparib in high-grade ovarian carcinoma
	PALB2	Stimulates strand invasion.	Gain	Cooperation of breast cancer proteins PALB2 and piccolo BRCA2 in stimulating homologous recombination
	SHFM1	Stimulates strand invasion.	Gain	Cooperation of breast cancer proteins PALB2 and piccolo BRCA2 in stimulating homologous recombination
	CDK12	Promotes homologous recombination.	Gain	Ovarian cancer-associated mutations disable catalytic activity of CDK12, a kinase that promotes homologous recombination repair and resistance to cisplatin and poly(ADP-ribose) polymerase inhibitors
	HELB	Inhibits DNA end resection.	Loss	HELB Is a feedback inhibitor of DNA end resection
	DYNLL1	Inhibits DNA end resection.	Loss	DYNLL1 binds to MRE11 to limit DNA end resection in BRCA1-deficient cells
	ATMIN	Transcriptionally activates DYNLL1.	Loss	DYNLL1 binds to MRE11 to limit DNA end resection in BRCA1-deficient cells
	MRE11	Promotes end resection and replication fork degradation.	Unclear^a^	Double-strand break repair-independent role for BRCA2 in blocking stalled replication fork degradation by MRE11
	PTEN	Controversial.	Loss	
	PIK3CA	PI3K controls DSB repair.	Gain	PI3K inhibition impairs BRCA1/2 expression and sensitizes BRCA-proficient triple-negative breast cancer to PARP inhibition
	PIK3CB	PI3K controls DSB repair.	Gain	PI3K inhibition impairs BRCA1/2 expression and sensitizes BRCA-proficient triple-negative breast cancer to PARP inhibition
	AKT1	PI3K controls DSB repair.	Gain	PI3K inhibition impairs BRCA1/2 expression and sensitizes BRCA-proficient triple-negative breast cancer to PARP inhibition
	POLQ	Catalyzes MMEJ, promoting cell survival in HR-deficient cells.	Gain	Polymerase theta inhibition kills homologous recombination deficient tumors
**Replication** **Fork**	PARP1	Promotes PARP trapping.	Loss	A genetic screen using the PiggyBac transposon in haploid cells identifies Parp1 as a mediator of olaparib toxicity
	PARG	Inhibits PARP parylation.	Loss	Selective loss of PARG restores PARylation and counteracts PARP inhibitor-mediated synthetic lethality
	PTIP (PAXIP1)	Promotes replication fork degradation.	Loss	Replication fork stability confers chemoresistance in BRCA-deficient cells
	MLL3	Promotes replication fork degradation.	Loss	Replication fork stability confers chemoresistance in BRCA-deficient cells
	MLL4	Promotes replication fork degradation.	Loss	Replication fork stability confers chemoresistance in BRCA-deficient cells
	EZH2	Promotes replication fork degradation.	Loss	EZH2 promotes degradation of stalled replication forks by recruiting MUS81 through histone H3 trimethylation
	SMARCAL1	Promotes replication fork degradation.	Loss	Restoration of replication fork stability in BRCA1- and BRCA2-deficient cells by inactivation of SNF2-family fork remodelers
	ZRANB3	Promotes replication fork degradation.	Loss	Restoration of replication fork stability in BRCA1- and BRCA2-deficient cells by inactivation of SNF2-family fork remodelers
	HLTF	Promotes replication fork degradation.	Loss	Restoration of replication fork stability in BRCA1- and BRCA2-deficient cells by inactivation of SNF2-family fork remodelers
	SLFN11	Stalls stressed replication forks.	Loss	Resistance to PARP inhibitors by SLFN11 inactivation can be overcome by ATR inhibition
	CHD4	Inhibits translesion synthesis.	Loss^1^	Resistance to therapy in BRCA2 mutant cells due to loss of the nucleosome remodeling factor CHD4
	RADX	Inhibits RAD51 at replication forks.	Loss	RADX promotes genome stability and modulates chemosensitivity by regulating RAD51 at replication forks
	TLK1	Stabilizes replication forks.	Gain	Tousled-like kinases stabilize replication forks and show synthetic lethality with checkpoint and PARP inhibitors
	TLK2	Stabilizes replication forks.	Gain	Tousled-like kinases stabilize replication forks and show synthetic lethality with checkpoint and PARP inhibitors
	PRIMPOL	Promotes repriming of stalled forks.	Gain	PRIMPOL-mediated adaptive response suppresses replication fork reversal in BRCA-deficient cells
**Other**	AR	Regulates HR genes.	Gain	Androgen receptor inhibitor enhances the antitumor effect of PARP inhibitor in breast cancer cells by modulating DNA damage response
	KRAS	RAS mutant cell lines are resistant to PARPi.	Gain	Rational combination therapy with PARP and MEK inhibitors capitalizes on therapeutic liabilities in RAS mutant cancers
	HRAS	RAS mutant cell lines are resistant to PARPi.	Gain	Rational combination therapy with PARP and MEK inhibitors capitalizes on therapeutic liabilities in RAS mutant cancers
	NRAS	RAS mutant cell lines are resistant to PARPi.	Gain	Rational combination therapy with PARP and MEK inhibitors capitalizes on therapeutic liabilities in RAS mutant cancers
	BRAF	RAS mutant cell lines are resistant to PARPi.	Gain	Rational combination therapy with PARP and MEK inhibitors capitalizes on therapeutic liabilities in RAS mutant cancers
	ARID1A	Unknown	Unclear	ARID1A Deficiency Impairs the DNA Damage Checkpoint and Sensitizes Cells to PARP Inhibitors, A quantitative chemotherapy genetic interaction map reveals factors associated with PARP inhibitor resistance
	GPBP1	Unknown	Loss	A quantitative chemotherapy genetic interaction map reveals factors associated with PARP inhibitor resistance
	TDG	Unknown	Loss	A quantitative chemotherapy genetic interaction map reveals factors associated with PARP inhibitor resistance
	RNF168	Multiple	Unclear	

^a^Phenotype appears to be BRCA-dependent.

Genes that are known or suspected to be linked to resistance to PARPi are shown. Resistance indicates whether a Gain or Loss of function has been either shown or inferred to lead to resistance to PARPi.

The oncoplot of the variants revealed mutations in potential resistance genes in nearly every tumor (Fig. 2a). There was frequent somatic copy number deletion in *BRCA1* and *BRCA2*, likely reflecting loss of heterozygosity events^57^. In the resistant tumors, alterations that may associate with resistance were seen across every patient except for P31 in genes including *SHLD2*, *RAD51*, *ATMIN*, *PTEN*, *PARG*, *MLL4*, *EZH2*, *SLFN11*, *AR*, *KRAS*, and *GPBP1*. We prioritized these genes by looking for mutations or copy number alterations that led to predicted loss or gain of function in PARPi resistant but not PARPi sensitive tumors. Four genes were identified as potential candidates mediating resistance including SHLD2 (FAM35A), PARG, MLL4 (KMT2B), and SLFN11. Of these, SHLD2 was the only one altered recurrently, with copy number deletion seen in three of the six resistant tumors. SHLD2 is a member of the shieldin complex, which mediates 53BP1-dependent non homologous end joining^20,58^. Loss of shieldin promotes HR, leading to resistance to PARPi. Here, all tumors with SHLD2 loss had g*BRCA1* mutations. The fact that SHLD2 loss was not observed in g*BRCA2* mutant tumors may be due to the fact that *BRCA2* acts downstream of shieldin, and thus shieldin loss would not confer resistance in g*BRCA2*+ tumors^59^.

To verify the copy number aberrations, we used an additional copy number caller Sequenza^60^, which confirmed copy number loss in the tumors of patients 23 and 34, but did not recapitulate the copy number loss seen in the tumor from patient 15. We then compared the gene expression of shieldin complex proteins between PARPi resistant and sensitive tumors (Fig. 2b, Supplementary Fig. S9). Expression of SHLD2 alone was 2.2x lower in PARPi resistant tumors (*p* = 0.003) and tumors with the lowest SHLD2 expression also had copy number loss in at least one of the callers. We repeated this analysis across the remainder of the 51 established resistance genes, regardless of mutation or copy number change. The expression of CDK12 and SMARCAL1 were both significantly higher in the sensitive tumors (both *p* = 0.03), but the average changes in gene expression were modest (1.2× and 1.3×, respectively) and therefore unlikely to indicate a general resistance mechanism (Supplementary Fig. S10). Thus, our analysis identified loss of SHLD2, either by copy number loss or reduced expression, as a potential mechanism for PARPi resistance in g*BRCA*+ tumors.

### Gene expression signatures are associated with both sensitivity and resistance to PARPi

**Fig. 3:**
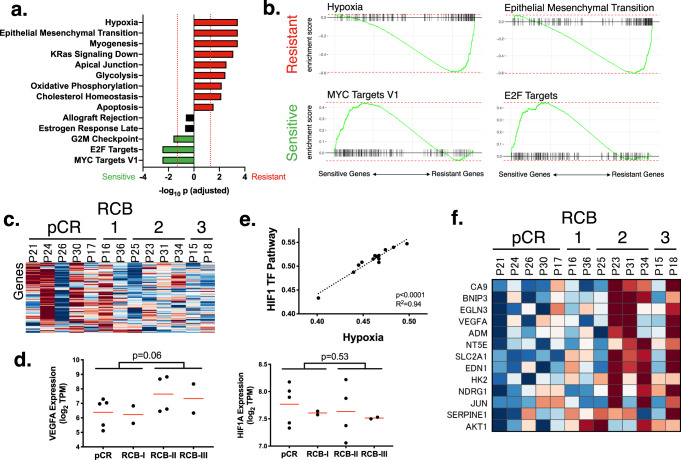
Pathways correlated with resistance. **a** This shows the association between Hallmarks pathways and response to talazoparib. Each bar stands for a pathway, and the height indicates the statistical significance as the -log_10_ of the adjusted *p*-value. The pathways that are associated with sensitivity to treatment are shown in green and those associated with resistance are red. **b** This contains the GSEA enrichment plots for pathways most strongly associated with resistance to talazoparib. Pathways that are higher in resistant tumors are in the top row, and the bottom contains the pathways higher in sensitive tumors. The genes are ranked such that the ones associated with sensitivity are on the left, and those with resistance are on the right. **c** This heatmap shows the expression of the most differentially regulated genes (leading edge) of the MYC Targets V1 pathway (rows) for each of the tumors (columns), organized by response. Warmer colors indicate higher expression, and cooler colors are lower expression. **d** These plots show the association of the expression of VEGFA (left) and HIF1A (right) with response. The mean expression is shown as a red line. *p*-values (unpaired two-sided Student’s *t* tests) are shown after comparing patients with no progression (pCR and RCB-I) against those that progressed (RCB-II and RCB-III). **e** This plot compares the scores of the hypoxia pathway (*x*-axis) against that with a HIF1 transcriptional network (*y*-axis). The *p*-value is calculated from a two-sided Pearson’s correlation coefficient test. **f** This heatmap shows the expression of the genes in the leading edge of the hypoxia pathway. The heatmap is arranged as in panel **c**.

To look more broadly for markers of resistance, we performed a pathway analysis. We used a pre-ranked GSEA (prGSEA) test (see Methods) on the Hallmarks database^61^ to identify the pathways that correlated with response. We found that sensitive tumors were associated with activation of E2F (adjusted *p* = 0.003), MYC (adjusted *p* = 0.003), and G2/M (adjusted *p* = 0.02) (Fig. 3a, b). This could represent more proliferative tumors or alternatively tumors with premature entry into S phase and replication stress that has been associated with response to PARPi^62^. In contrast, resistant tumors had high predicted activity of hypoxia (adjusted *p* = 0.0004), EMT (adjusted *p* = 0.0004), altered metabolism (glycolysis adjusted *p* = 0.003, oxidative phosphorylation adjusted *p* = 0.007, cholesterol homeostasis adjusted *p* = 0.007), along with several other pathways.

To verify the association between MYC targets and sensitivity to talazoparib, we extracted the genes from the leading edge of the signature (i.e., the genes with the largest change in expression) and visualized their expression in a heatmap (Fig. 3c). This confirmed that nearly all the tumors that achieved pCR, with the exception of P26, had high expression of MYC target genes, while the tumors with RCB-II and -III had lower expression across those genes.

In resistant tumors, the hypoxia gene expression signature was predicted to be active. As an initial validation of this result, we scored the tumors with a validated hypoxia signature^63^ and found that its scores were highly correlated with the Hallmarks signature from our analysis (Supplementary Fig. S11). Next, we asked whether gene expression of known mediators of hypoxia, HIF1A (a downstream transcriptional regulator of a hypoxia response) and VEGF-A (a hypoxia responsive growth factor) (reviewed in^64^) had concomitant changes (Fig. 3d, Supplementary Fig. S12). VEGF-A was expressed higher in resistant tumors, although it did not achieve statistical significance (*p* = 0.06). Expression of HIF1A was not associated with response (*p* = 0.53). This could be because HIF1A is regulated post-translationally by ubiquitination, and its activity may not correlate with its gene expression levels. Therefore, we examined whether expression of HIF target genes^65^ correlated with the hypoxia signature and found that HIF target genes were upregulated in resistant tumors (*p* < 0.0001) (Fig. 3e). The expression levels of HIF1 targets (seen on the leading edge of a GSEA analysis), including VEGF-A, were highly upregulated in 4 out of the 6 most aggressive tumors (Fig. 3f). This provides evidence that hypoxia is a common feature in talazoparib-resistant tumors.

### An EMT signature and stem cell markers are correlated with resistance

**Fig. 4:**
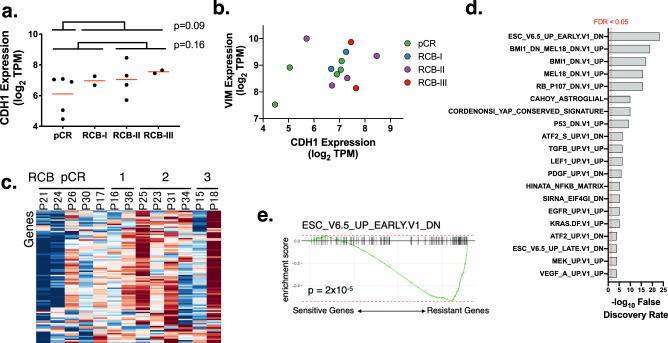
A stem cell transcriptional program was associated with response. **a** The gene expression (log_2_ TPM) of CDH1 is shown on the *y*-axis. Each dot is a tumor, grouped according to the response to talazoparib. The red lines indicate mean expression. The *p*-values (by unpaired two-sided Student’s *t* test) are shown for comparisons between patients with pCR vs other patients, as well as a comparison between pCR and RCB-I, and RCB-II and RCB-III. **b** The gene expression for CDH1 (*x*-axis; log_2_ TPM) and VIM (*y*-axis; log_2_ TPM) are shown for tumors colored by response. **c** The rows in the heatmap contain the genes in the leading edge of the EMT gene expression signature, and the columns are samples organized according to response. Warm colors indicate high expression of the genes. **d** The bars in this plot show the significance (as -log_10_ of the false discovery rate) of the top 20 most significant oncogenic pathway (rows). The dotted red line shows the cutoff for a 5% false discovery rate. **e** The enrichment plots for the oncogenic pathway that is most significantly associated with response.

In addition to the hypoxia signature, the pathway analysis also showed an equally strong association of epithelial mesenchymal transition (EMT) with resistance to talazoparib (Fig. 3a), which was also previously seen in PDX models of small cell lung cancer^66^. To corroborate the EMT signature, we evaluated tumors for the expression of CDH1, the primary cadherin responsible for epithelial cell adhesion that is suppressed in EMT^67,68^. Surprisingly, we found that resistant tumors had higher, rather than lower, levels of CDH1 expression, although the differences were not statistically significant (*p* = 0.16) (Fig. 4a). Next, we examined tumors for expression of both CDH1 (an epithelial state marker) and vimentin (VIM, a mesenchymal state marker) but did not see a clear distinction between canonical epithelial cells (high CDH1 and low VIM) and mesenchymal cells (low CDH1 and high VIM)^69^ (Fig. 4b). Therefore, despite the correlation between PARPi resistance and an EMT pathway signature, resistant tumors did not exhibit classic EMT markers.

Because the EMT is associated with a number of phenotypes, we investigated the possibility that activation of a subset of the EMT program may be responsible for the high scores in the gene expression signature, even if an EMT itself is not achieved. We visualized the expression of the 93 genes on the leading edge of the prGSEA analysis (i.e., the most differentially expressed genes from the EMT gene set) and saw that they are more highly expressed in the resistant tumors (Fig. 4c). To understand their function, we performed a GATHER analysis^70^ with a collection of oncogenic gene sets, which is more comprehensive than Hallmarks, from MSigDB. This revealed 63 pathways that are significantly (false discovery rate (FDR) < 5%) enriched in the subset of the EMT program (Fig. 4d). The most significant one was associated with genes upregulated in embryonic stem cells (ESC_V6.5_UP_EARLY.V1_DN; FDR = 10^−23^). The fact that the EMT signature was not correlated with EMT markers, but was associated with a stem cell signature, suggests that the Hallmark EMT signature was evincing an EMT-associated stem cell program^71^. To determine whether the stem cell program was seen in patient tumors, we scored it on the tumors using prGSEA and found that it was significantly upregulated in resistant tumors (*p* = 2 × 10^−5^) (Fig. 4e).

## Discussion

We previously demonstrated the efficacy and reduced toxicity of talazoparib as a single-agent neoadjuvant therapy for previously untreated g*BRCA*+ cancers^13^. However, not all patients demonstrated benefit as indicated by complete responses to neoadjuvant therapy. Thus, biomarkers of both sensitivity and resistance to PARPi are needed to identify patients most likely to benefit, and resistance remains a critical issue that must be addressed to achieve durable responses in a greater number of patients. Prior studies to determine mechanisms underlying sensitivity and resistance to PARPi have been performed predominantly in cell culture and genetically engineered mouse models, and little is known about the emergence of resistance in the clinical setting of breast cancer, particularly in the up-front neoadjuvant setting.

**Fig. 5:**
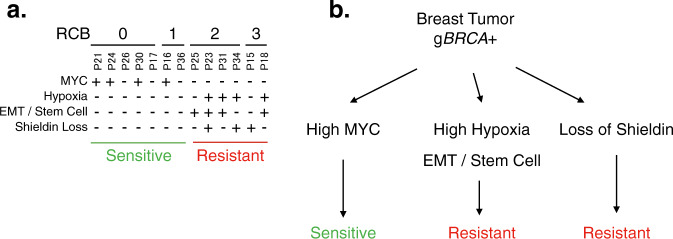
Model. **a** This table shows each of the tumors in the trial, grouped by RCB status. The tumors predicted to have an activated *Hypoxia* pathway, based on the pathway signature and expression of HIF targets, are indicated in the first row with a “+”. The *EMT / Stem Cell* status is determined from the expression of the leading edge EMT genes. *Shieldin Loss* reflects the concomitant loss of copy number and expression of SHLD2. **b** In this cohort of g*BRCA* mutant breast cancer patients, loss of shieldin, high hypoxia signature, or high EMT/Stem Cell signature is predictive of resistance.

To identify correlates of resistance, we performed an extensive genomic and transcriptomic analysis of treatment-naïve g*BRCA*+ breast cancer tumors treated with neoadjuvant talazoparib monotherapy. This study establishes, for the first time, correlates of sensitivity and resistance to a single agent PARPi in a treatment naïve neoadjuvant breast cancer clinical setting. It reveals potential resistance mechanisms including copy number loss coupled with low expression of the shieldin complex member SHLD2, or a gain of a hypoxia, or EMT/stem cell signature (Fig. 5). The potential role that the shieldin complex plays in promoting resistance to PARPi has been well established in pre-clinical studies. Here, all resistant tumors exhibited either loss of shieldin, gain of hypoxia signature, or gain of an EMT/stem cell signature. Potential biomarkers of response included MYC and E2F1 signatures.

The link between EMT, cancer stem cells, and resistance to chemotherapies is well established^72^ and reported for PARPi as well^73^. However, the connection between EMT and PARP function remains an intense area of investigation, as PAR appears to be present in epithelial adherens junctions^74^, and PARP1 has both positive^75,76^ and negative^77,78^ associations with TGF-ß signaling, a potent inducer of an EMT^79^ that may be context dependent.

In contrast to EMT, the connection between PARPi and hypoxia has been explored more deeply, although with mixed success thus far in the clinic. Under a severe hypoxic condition (0.5% oxygen), cells are more sensitive to PARPi due to the synthetic lethality induced by decreased expression of HR proteins^80^. However, the opposite is seen in moderate hypoxic conditions (2% oxygen) where a hypoxia signature predicted resistance to a PARPi^81^ due to decreased ROS-induced DNA damage^82^. In a breast cancer mouse model, hypoxic cells were shown to contribute to PARPi resistance, and targeting tumors with a combination of hypoxia and PARP inhibitors leads to synergistic toxicity. However, the clinical trial results are inconsistent, with phase 2 trials of olaparib and cederinib showing an increased progression free survival^83,84^ whereas the phase 3 trial failed to confirm benefit^85^. As a possible explanation, these studies may be confounded by the pleiotropic effects of cediranib, which can inhibit both VEGFR and decrease expression of HR proteins^86^. Furthermore, the trials were done in an unselected patient population and the number of patients with a pre-existing tumor hypoxia signature may have been sufficiently low as to confound the outcomes analysis. In a number of other disease contexts, including myocardial ischemia, stroke, and traumatic brain injury, PARPi have been observed to be protective against ischemic cell death (reviewed in^87–89^). Unfortunately, the mechanism has not yet been established, and it is not known whether a similar process might be activated in hypoxic tumors and render them refractory to PARPi treatment. Nevertheless, the observation that a hypoxia/stem cell signature exists in the majority of PARPi resistant tumors prior to therapy argues for additional studies to understand whether hypoxia can induce a PARPi resistant state, to elucidate underlying mechanisms, and to determine whether targeting hypoxia will increase the benefit of PARPi in patients with a pre-existing hypoxia signature.

Markers of high MYC activity were frequently observed in PARPi sensitive tumors. The relationship between MYC and DNA damage has been well-documented and controversial. Activated MYC can induce chromosomal damage^90,91^ including defects in double-stranded break repair^92,93^. Another consequence of activated MYC, increased proliferation, may enhance sensitivity to PARPi via PARP trapping, whose toxicity has been shown to be specific to S-phase^94^. In contrast, it has also been reported that MYC can facilitate DNA repair by inducing the expression of genes involved in HR, including BRCA1^95^, which may lead to PARPi resistance^96^. The impact of MYC is likely to be context dependent and the relationship between MYC activation and DNA repair in the human tumor environment requires further investigation.

There are several major limitations in this neoadjuvant study of g*BRCA*+ tumors. 1) The sample size was limited. We profiled 18 tumors, and only 13 could be used to interrogate the biology of the cancer cells. A prior study of a larger cohort of ovarian cancer patients found ABCB1 fusions in 8% of patients and *BRCA*1/2 reversions in another 5%, suggesting that resistance mechanisms are heterogeneous and likely to be found at low frequencies^97^. 2) We profiled pre-treatment samples and therefore can only detect intrinsic mechanisms of resistance and sensitivity. Longitudinal profiling will be needed to identify resistance mechanisms that are acquired during the course of treatment. The majority of patients had insufficient tumor at surgery following completion of talazoparib therapy therefore limiting detailed longitudinal analysis. 3) Our bulk genomic assays may not have sufficient sensitivity to identify mechanisms that are present in pre-treatment samples at low clonality levels, such as subclones with *BRCA* reversions^27,30^. Deep sequencing, single cell sequencing, or ctDNA profiling may facilitate the identification of genetic or transcriptomic events that occur subclonally. 4) Our exome sequencing data may not reveal the vast majority of possible genomic re-arrangements, leaving the possibility that gene fusions contributing to PARPi resistance and sensitivity were missed. 5) We did not interrogate non-coding RNAs, which have previously been suggested to mediate resistance^98^. 6) We considered only the cancer cells and did not profile the microenvironment, which can impact the efficacy of PARPi. As one example, it was recently shown that PARPi leads to DNA damage that triggers the cGAS/STING pathway, type I interferon signaling, and then cytotoxic T cell function; resulting in tumor clearance^99–101^. Finally, 7) this is an observational study of a single data set, and follow-up studies will be needed to determine whether our findings causally lead to resistance, and thus, would be viable targets for therapy.

In conclusion, our findings demonstrate that a number of biomarkers for sensitivity to single-agent talazoparib can be identified in treatment-naïve g*BRCA*+ samples. This supports previous findings showing that intrinsic activities in cancer cells have an impact on the clinical response to PARPi. The markers we identified provides a potential explanation for the high rates of relapse seen in patients treated with PARP inhibitors, several of which exhibited copy number loss of SHLD2. These results predict that defects in the shieldin complex may be a relatively frequent mechanism of resistance to single agent PARPi in patients, and if verified in larger cohorts, may have a role as a biomarker to exclude patients from therapies that will not be effective. Other resistant patients exhibited a hypoxia signature. As this manuscript was under review, a paper was published showing the efficacy of a combinatorial treatment targeting PARP and hypoxia in a breast cancer PDX model^82^, demonstrating the potential for developing therapeutic strategies to overcome this resistance. Further mechanistic studies are needed to understand the signaling networks engaged by these biomarker pathways to select viable therapeutic targets, and translational studies are needed to develop clinical tests and treatments to reverse resistance.

## Methods

### Patient cohort

The data was derived from a neoadjuvant trial of single agent talazoparib^13^. The trial was approved by the institutional review board under protocol 2014-0045, and each participant provided written informed consent. Briefly, twenty patients with clinical stage I-III breast cancer and an identified germline *BRCA1* or *2* pathogenic variant were enrolled. Patients received 6 months of single agent talazoparib starting at a dose of 1 mg orally daily. Surgery was scheduled within 5 weeks of the last dose of talazoparib. Final pathology was determined by a dedicated breast pathologist at our institution, and we used residual cancer burden as a measure of response^102^.

### Genomic DNA, library prep, and capture

Genomic DNA was quantified by Picogreen (Invitrogen) and quality was accessed using Genomic DNA Tape for the 2200 Tapestation (Agilent). DNA from each sample (100–500 ng of genomic DNA) was sheared by sonication with the following conditions: Peak Incident Power 175, Duty Cycle 20%, Intensity 5, Cycles per Burst 200, and 120 s using Covaris E220 instrument (Covaris). To ensure the proper fragment size, samples were checked on TapeStation using the DNA High Sensitivity kit (Agilent). The sheared DNA proceeded to library prep using the KAPA library prep kit (KAPA) following the “with beads” manufacturer protocol. Briefly, this protocol consists of 3 enzymatic reactions for end repair, A-tailing and Adaptor ligation, followed by barcode insertion by PCR using KAPA HiFi polymerase (6 cycles). PCR primers were removed by using a 1.8x volume of Agencourt AMPure PCR Purification kit (Agencourt Bioscience Corporation). At the end of the library prep, samples were analyzed on TapeStation to verify correct fragment size and to ensure the absence of extra bands. Samples were quantified using the KAPA qPCR quantification kit. Equimolar amounts of DNA were pooled for capture (2–6 samples per pool). We used whole-exome biotin labeled probes from Roche Nimblegen (Exome V3) and followed the manufacturer’s protocol for the capture step. Briefly, DNA was pooled (2–6 samples), dried out and after addition of the capture reagents and probes, samples were incubated at 47 °C on a thermocycler with a heated lid (57 °C) for 64–74 h. The targeted regions were recovered using streptavidin beads and the streptavidin-biotin-probe-target complex was washed and another round of PCR amplification was performed according to the manufacturer’s protocol. The quality of each captured sample was analyzed on TapeStation using the DNA High Sensitivity kit and the enrichment was accessed by qPCR using specific primers designed by Roche Nimblegen. The cutoff for the enrichment was 50-fold minimum.

### Mutation data analysis

The captured libraries were sequenced on a HiSeq 2000 (Illumina Inc., San Diego, CA, USA) on a version 3 TruSeq paired end flowcell at a cluster density between 700–1000 K clusters/mm^2^. Sequencing was performed for 2 × 100 paired-end reads with a 7 nt read for indexes using Cycle Sequencing v3 reagents (Illumina). The resulting BCL files containing the sequence data were converted into “.fastq.gz” files and individual libraries within the samples were demultiplexed using CASAVA 1.8.2 with no mismatches.

We called single nucleotide variants (SNVs) using a pipeline^103^ implemented in the BETSY system^104^. Starting with the FASTQ files, we trimmed adapter and low-quality sequence using Trimmomatic^105^. Next, we aligned the trimmed reads to human reference assembly hg19 using BWA^106^ and flagged duplicated reads using Picard^107^. We realigned indels and recalibrated base quality scores using GATK^108^. Then, we called somatic variants using a consensus of six callers (MuSE^109^, MuTect^110^, MuTect2, SomaticSniper^111^, Strelka2^112^, and VarScan2^113^). For the Strelka analysis, we skipped the depth filter and set minPruning = 3 in MuTect2 to speed up computation^114^. To confirm the identities of the germline and tumor samples, we used GATK to call variants on the samples and confirmed that the tumor samples shared mutations with its cognate germline. We annotated variants with Annovar^115^ and SnpEff^116^, and filtered for nonsynonymous changes supported by at least 2 callers, 20 reads, and 5% variant allele frequency. We also annotated the mutations using Variant Effect Prediction^117^ and selected the most severe consequence per variant. Synonymous, intergenic, intronic and non-coding transcript mutations were removed.

We estimated copy number profiles using the FACETS algorithm^35^. We varied the parameters used for FACETS (critical value from 100–500, nbhd from 100–500, and nhet from 15–50). Following a manual examination of the log-ratio and log-odds-ratio plots, we decided to use the models with parameters critical value 300, nbhd 250, and nhet 30 because they had the most plausible models across the samples. We also estimated copy number profiles using Sequenza^60^ with default parameters and manually selected the most plausible of the alternative models. Finally, we estimated tumor purity and ploidy with ABSOLUTE^36^ with a sigma_p 0.02, max_sigma_h 0.02, max.non.clonal 0.1, min.ploidy 0.95, and max.ploidy 10.

We performed mutational signature analysis using the DeconstructSigs^49^ and SigMA^48^ R packages. We ran DeconstructSigs on the mutations found by at least 2 callers and supported by at least 20 reads and 5% VAF using the *signatures.nature2013* reference with the *exome2genome* mapping. In contrast to the set of mutations above, we did not filter for nonsynonymous changes here. We ran SigMA using the same filtering criteria as DeconstructSigs on the *breast* tumor type with the *seqcap* data option.

We calculated the tumor mutation burden as the number of non-synonymous mutations per megabase of targeted exome, as determined from the BED files downloaded from the Roche Nimblegen website. We calculated the weighted Genome Instability Index^118^ as the percent of the genome with either gain or loss of copy number relative to the median copy number of the sample. This was calculated separately for chromosomes 1–22 and then averaged so that the score was not biased by chromosome length.

We calculated the Large-Scale State Transition (LST) and Telomeric Allelic Imbalance (TAI) scores from the FACETS allele-specific copy number predictions described above. We implemented the LST scores^52^ following bio-protocol (10.21769/BioProtoc.814). Briefly, we split the genome into segments with identical copy number profiles (i.e., the same number of major and minor alleles). Then, we counted the number of segments that were at least 10 megabases long that were within 3 megabases of another 10 megabase segment with a different copy number profile. Although the original algorithm smoothed the copy number profiles of short (<3 megabase) segments, we dropped the short segments entirely, as this had only a minor impact on the scores.

We calculated TAI scores^54^. To do this, we looked for regions at the end of the chromosome where the major and minor alleles had different numbers. If this imbalance originated from the telomeres and did not extend to the centromeres (extracted from the UCSC Genome Browser), we increased the TAI score. We made one alteration to the original algorithm and ignored the p-arms of chromosomes 13, 14, 15, and 22. Because these chromosomes were acrocentric, the p-arms lacked coverage in our whole-exome sequencing panel, and thus, we were not able to obtain estimates of copy number there.

### Gene expression data analysis

We used STAR^119^ to align RNA-sequencing reads and HTSeq-count^120^ to quantify gene-level counts. We performed pathway analysis using a pre-ranked GSEA implemented in fGSEA^121^, after ranking the genes by differential expression statistics from DESeq2^122^. We used gene sets found in MSigDB v6.2^61^. We annotated gene sets using GATHER^70^.

### Reporting summary

Further information on research design is available in the Nature Research Reporting Summary linked to this article.

## Supplementary information

Supplementary Information

Reporting Summary

## Data Availability

RNA-seq data described in this study are deposited in the Gene Expression Omnibus database under accession GSE160568. The raw RNA-Seq and whole-exome sequencing data are available under controlled access from the European Genome-phenome Archive under accession EGAD0000100827.
