# Supplementary material for: Identification of biomarkers of response to preoperative talazoparib monotherapy in treatment naïve gBRCA+ breast cancers
Source: NPJ Breast Cancer. 2022 May 10;8:64. doi: 10.1038/s41523-022-00427-9 (PMC9090765; doi:10.1038/s41523-022-00427-9)
Supplement: Supplementary file 1 — Supplementary Information [file 41523_2022_427_MOESM1_ESM.pdf]

Supplementary Data

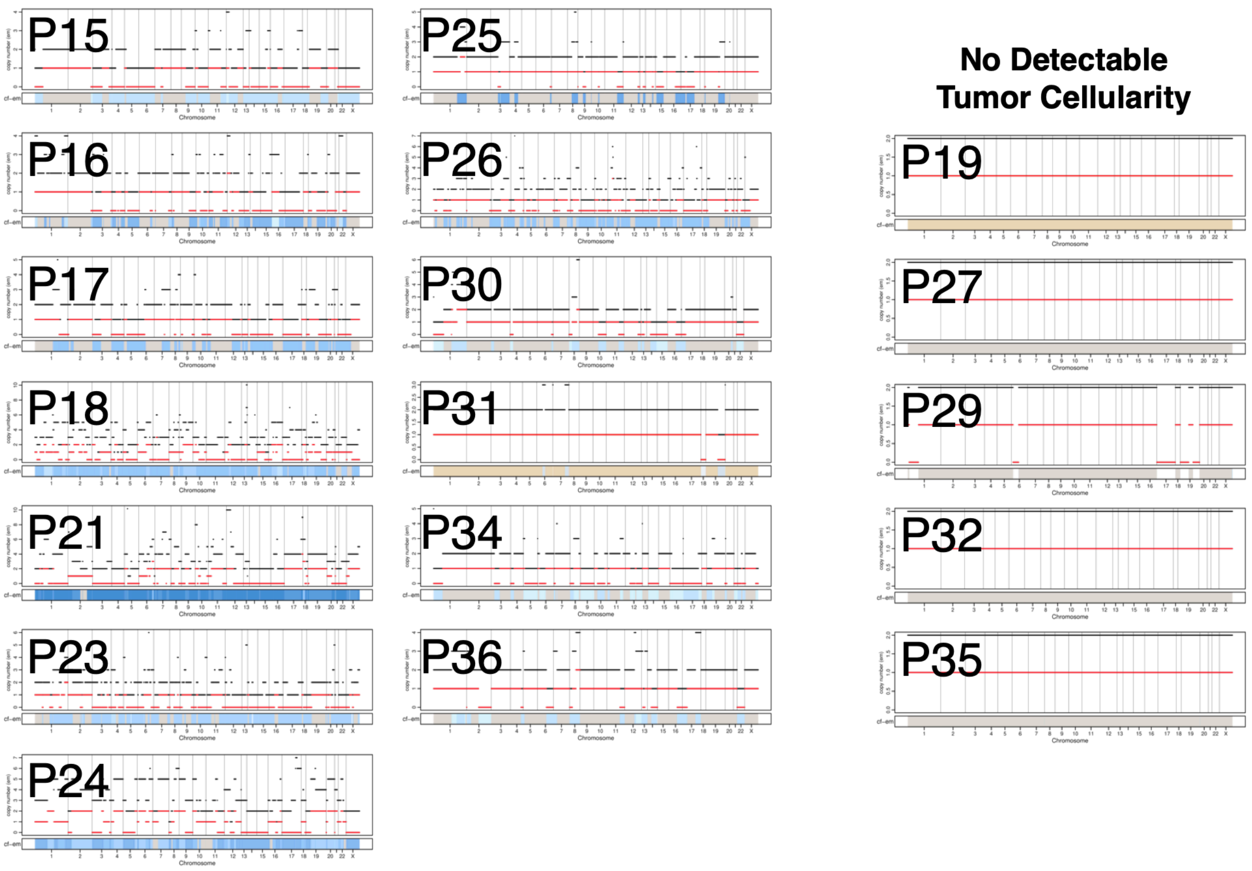

Supplementary Figure 1. The estimated genomic copy number profiles from FACETS are shown. The five tumors predicted by FACETS to have no tumor cell cellularity are shown on the right.

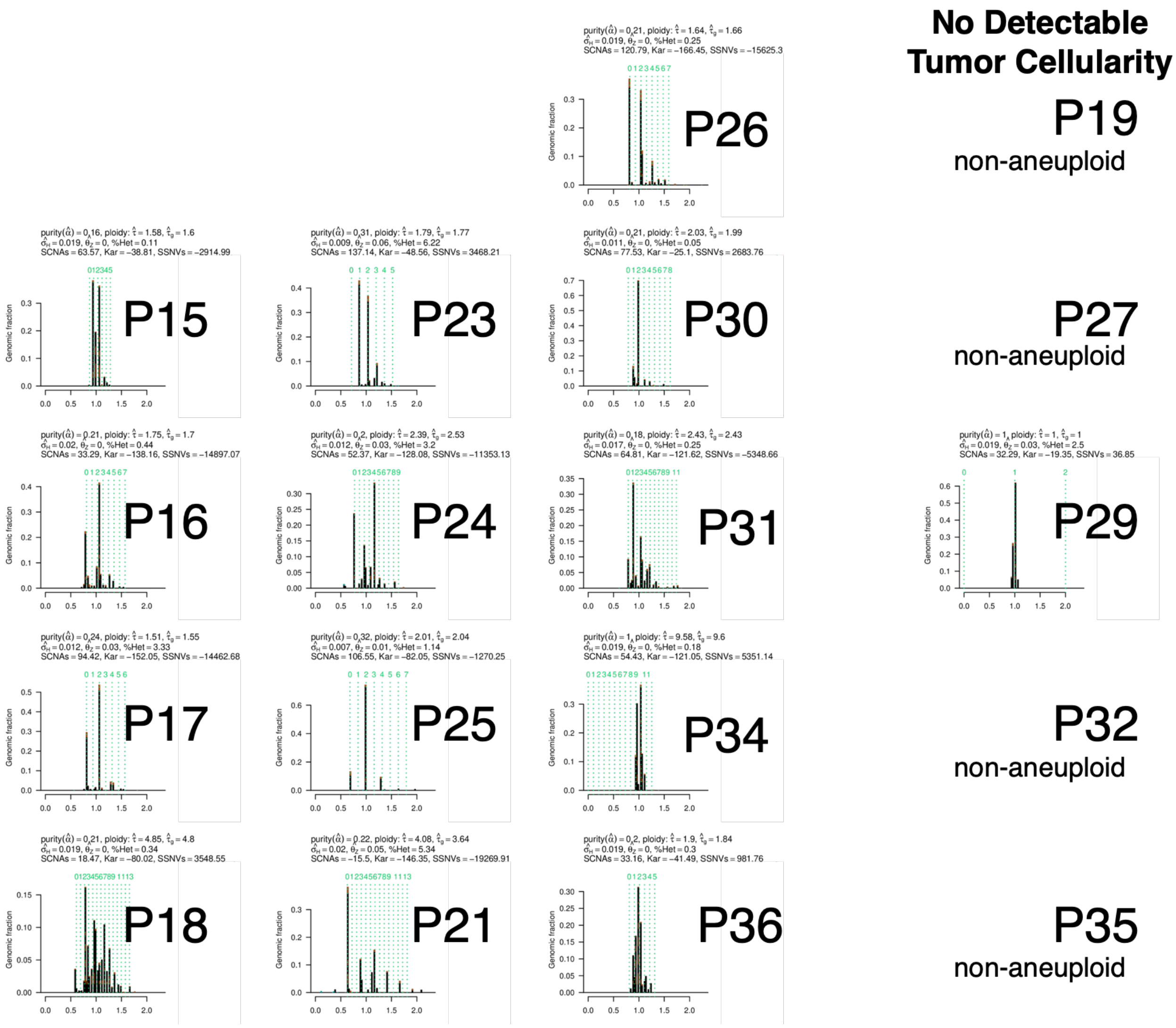

Supplementary Figure 2. Genomic fraction plots from ABSOLUTE are shown. The five tumors predicted by FACETS to have no tumor cell cellularity are shown on the right. Four of these tumors were predicted by ABSOLUTE to be non-aneuploid and no calls were generated.

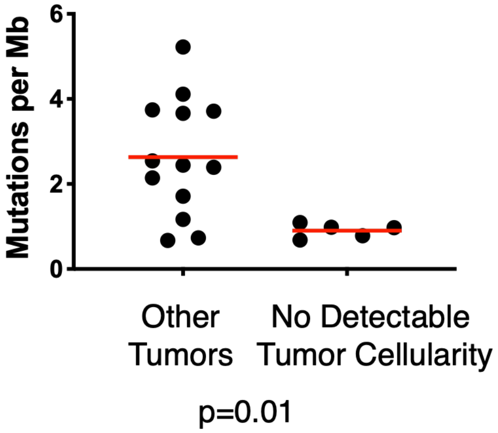

Supplementary Figure 3. Tumor mutation burden (y-axis) of tumors where FACETS was able to generate copy number calls (left) and those where it could detect no copy number changes (right).

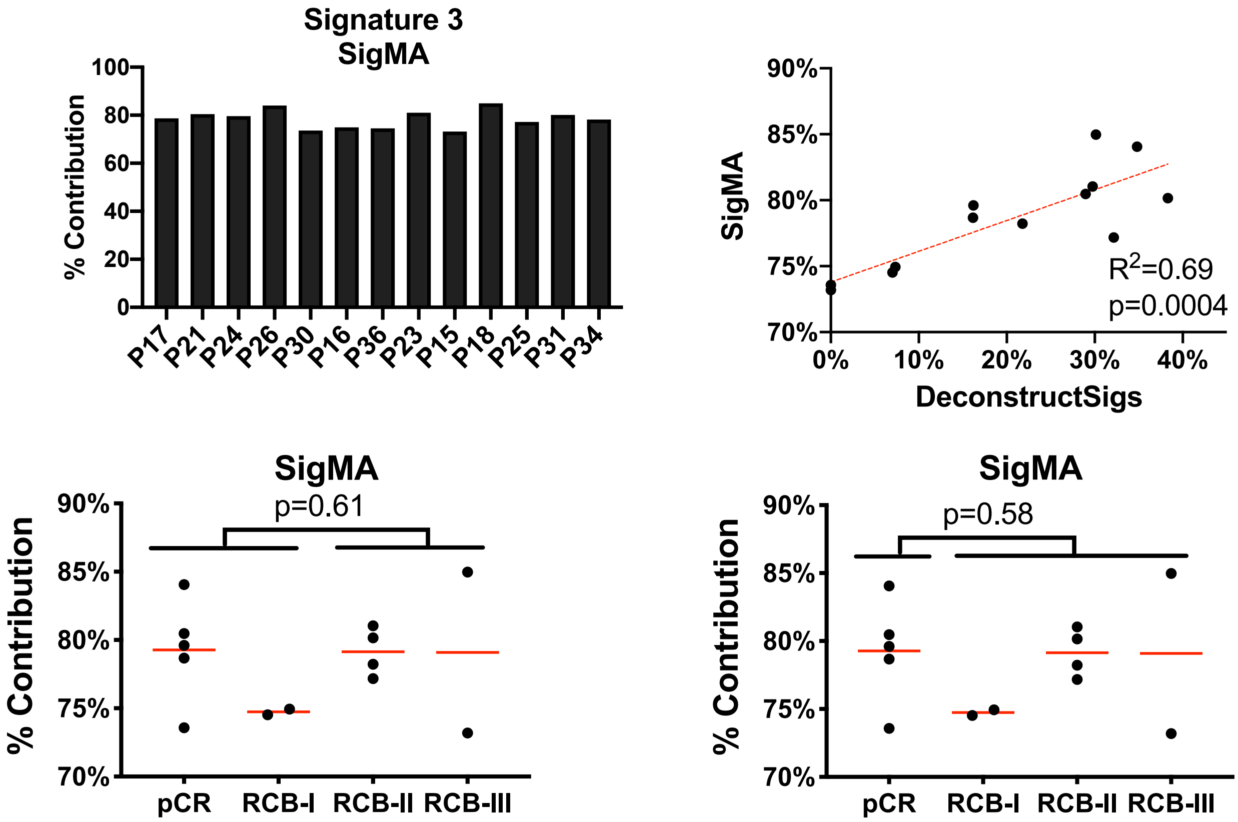

Supplementary Figure 4. (top left) The contribution of signature 3 predicted by SigMA (y-axis) is shown for each of the tumor samples (each column). (top right) This scatter-plot shows the correlation between the signature 3 scores predicted by DeconstructSigs (x-axis) and SigMA (y-axis). A linear regression line is shown in red. (bottom left) The SigMA scores are discretized based on the RCB scores. Sensitive tumors are defined as those achieving pCR or RCB-I, and resistant ones are RCB-II or RCB-iii. (bottom right) SigMA scores for tumors with pCR are compared against those that did not achieve pCR.

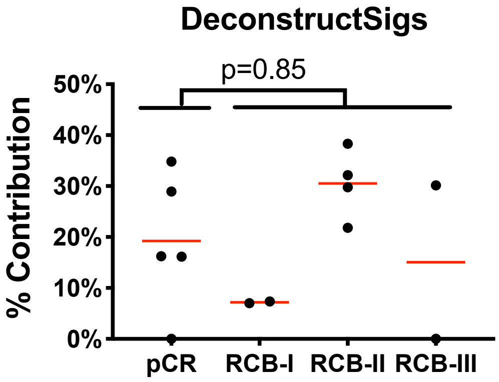

Supplementary Figure 5. This shows the contribution from signature 3 (y-axis) as predicted by DeconstructSigs as in Figure 1c. The p-value shows the significance of the comparison between tumors with pCR and others.

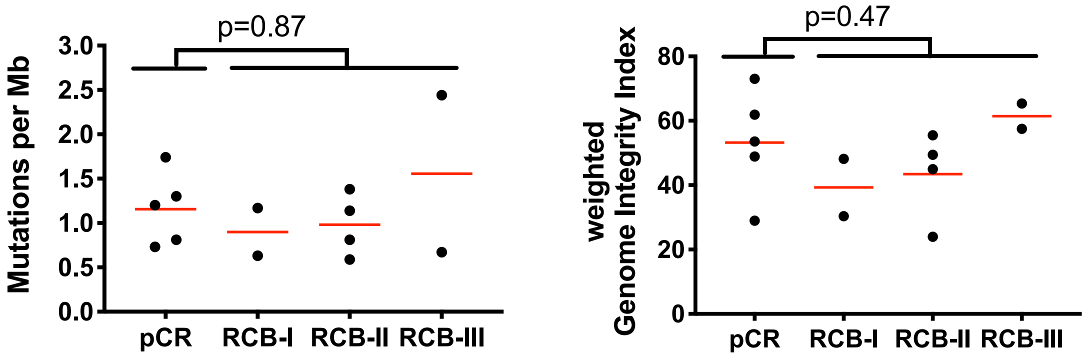

Supplementary Figure 6. These plots show the tumor mutation burden (left) and weighted Genome Integrity Index (right) as in Figures 1d and 1e. The p-values show the significance of the comparisons between tumors with pCR and others.

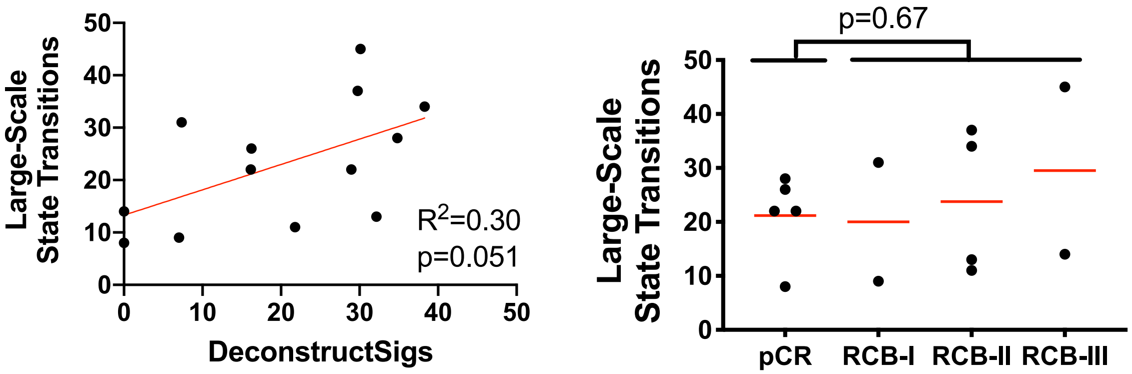

Supplementary Figure 7. (left) This scatterplot compares the percent contribution from signature 3 (x-axis) predicted by DeconstructSigs against the number of large-scale state transitions (y-axis) for each of the tumors (points). (right) The number of large-scale state transitions for each tumor is shown, separated by response. The p-value shows the significance of the comparison between the tumors that achieved pCR against those that did not.

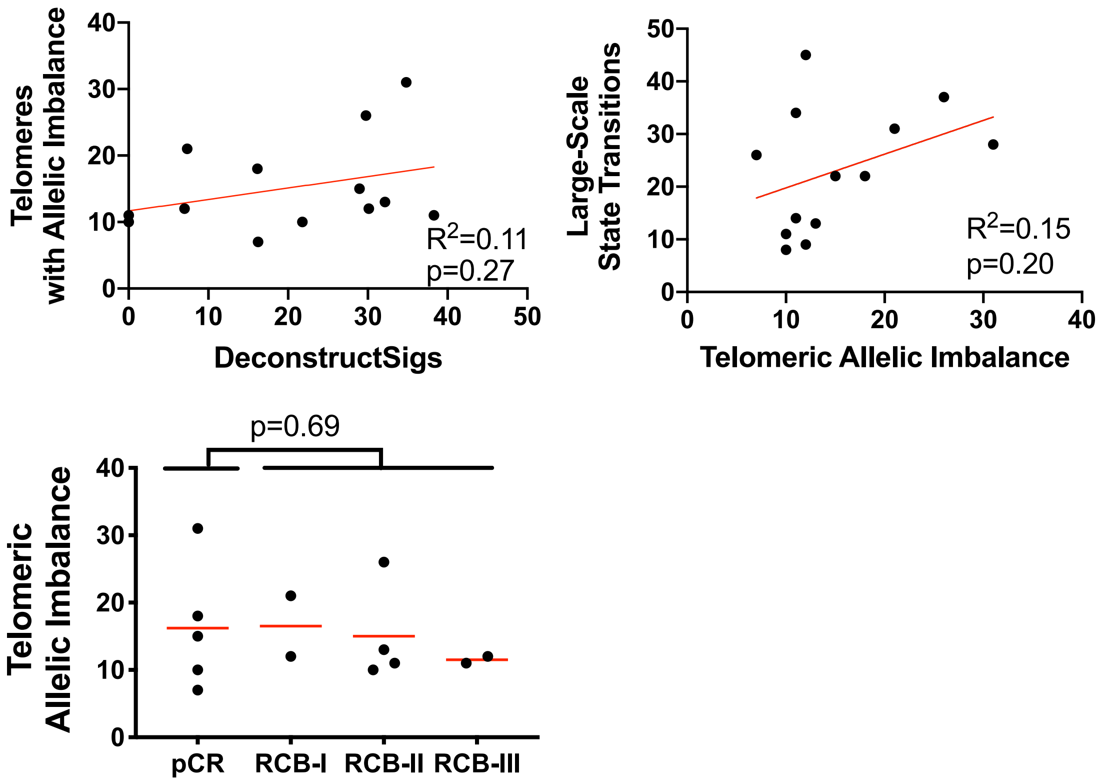

Supplementary Figure 8. (top left) This scatter plot compares the percent contribution from signature 3 as predicted by DeconstructSigs (x-axis) against the telomeric allelic imbalance score (y-axis) for each of the tumors (points). (top right) This shows the comparison between the telomeric allelic imbalance scores (x-axis) and the number of large-scale state transitions (y-axis). (bottom left) The telomeric allelic imbalance scores (y-axis) are shown for each of the tumors, separated by RCB response. The p-value shows the significance of the difference between the scores for the tumors that achieved pCR and those that did not.

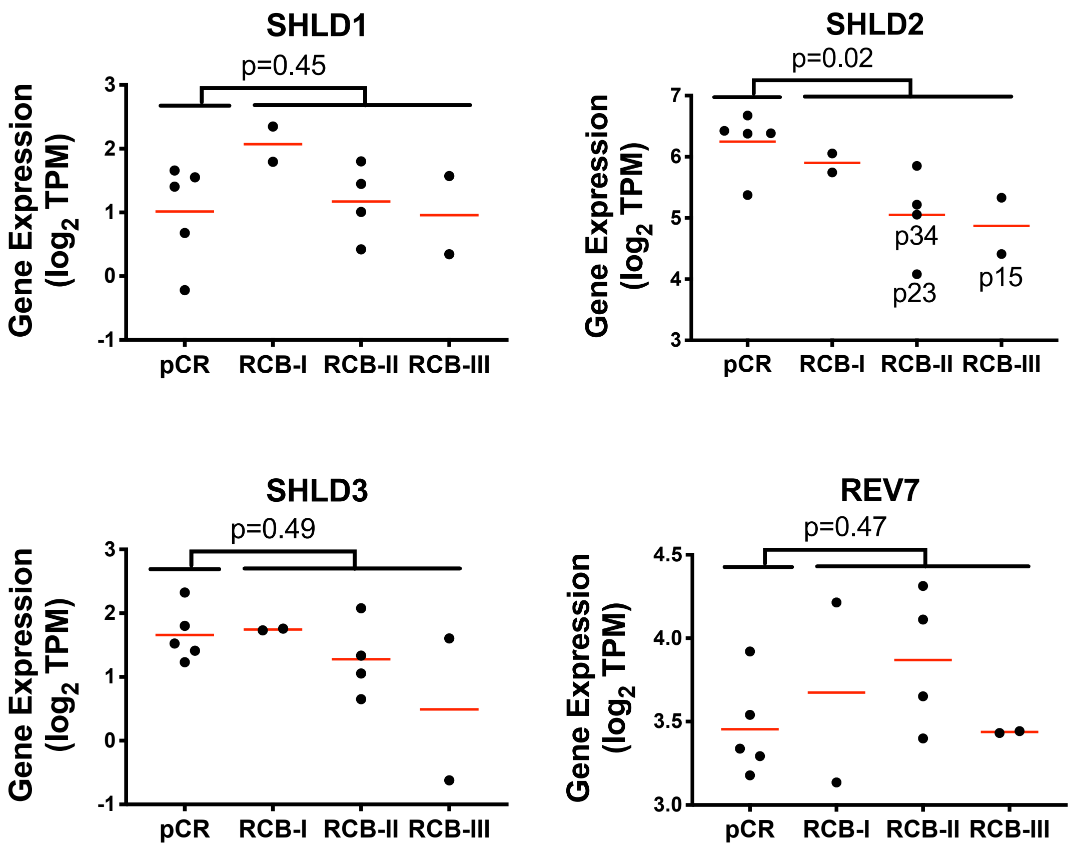

Supplementary Figure 9. These plots show the gene expression values (y-axis) for the Shieldin complex members for each of the tumors partitioned by RCB response. The p-values indicate the significance of the difference in expression between the tumors that achieved a pCR against those that did not.

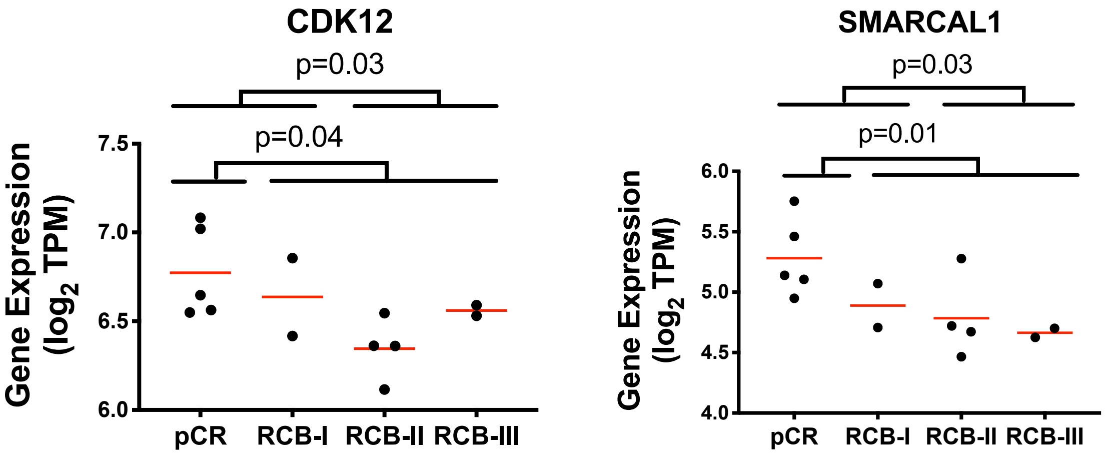

Supplementary Figure 10. Gene expression for *CDK12* and *SMARCAL1* are shown on the Y-axis. Individual patients (dots) are grouped according to RCB response. Mean expressions indicated by red lines. The p-values are calculated from unpaired two-sided Student's t-tests.

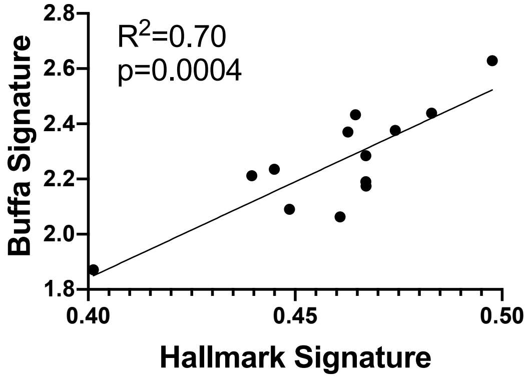

Supplementary Figure 11. The scores for the Hallmarks hypoxia signature for the 13 tumors samples (points) are shown on the x-axis, and the scores for the Buffa et al. hypoxia signature are shown on the y-axis.

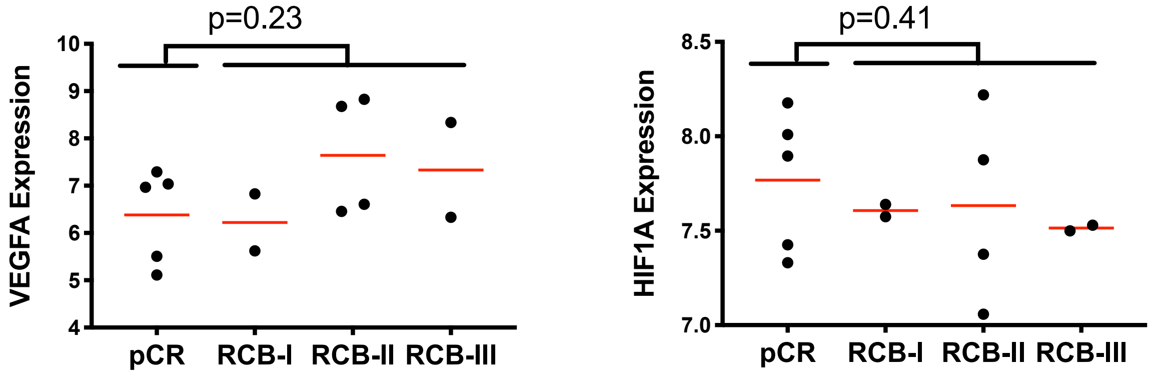

Supplementary Figure 12. The gene expression (y-axis, log_2 TPM) of VEGFA (left) and HIFA1 (right) is shown for each of the tumors, partitioned by RCB response. The p-values indicate the significance of the difference in expression between the tumors that achieved pCR against those that did not.
