# Supplementary material for: Identification of biomarkers of response to preoperative talazoparib monotherapy in treatment naïve gBRCA+ breast cancers
Source: NPJ Breast Cancer. 2022 May 10;8:64. doi: 10.1038/s41523-022-00427-9 (PMC9090765; doi:10.1038/s41523-022-00427-9)
Supplement: Supplementary file 2 — Reporting Summary [file 41523_2022_427_MOESM2_ESM.pdf]

nature portfolio

Corresponding author(s): Chang

Last updated by author(s): Mar 30, 2020

Reporting Summary

Nature Portfolio wishes to improve the reproducibility of the work that we publish. This form provides structure for consistency and transparency in reporting. For further information on Nature Portfolio policies, see our Editorial Policies and the Editorial Policy Checklist.

Statistics

For all statistical analyses, confirm that the following items are present in the figure legend, table legend, main text, or Methods section.

n/a	Confirmed	
☐	☒	The exact sample size (*n*) for each experimental group/condition, given as a discrete number and unit of measurement
☐	☒	A statement on whether measurements were taken from distinct samples or whether the same sample was measured repeatedly
☐	☒	The statistical test(s) used AND whether they are one- or two-sided<br>*Only common tests should be described solely by name; describe more complex techniques in the Methods section.*
☐	☒	A description of all covariates tested
☐	☒	A description of any assumptions or corrections, such as tests of normality and adjustment for multiple comparisons
☐	☒	A full description of the statistical parameters including central tendency (e.g. means) or other basic estimates (e.g. regression coefficient) AND variation (e.g. standard deviation) or associated estimates of uncertainty (e.g. confidence intervals)
☐	☒	For null hypothesis testing, the test statistic (e.g. *F*, *t*, *r*) with confidence intervals, effect sizes, degrees of freedom and *P* value noted<br>*Give P values as exact values whenever suitable.*
☒	☐	For Bayesian analysis, information on the choice of priors and Markov chain Monte Carlo settings
☒	☐	For hierarchical and complex designs, identification of the appropriate level for tests and full reporting of outcomes
☒	☐	Estimates of effect sizes (e.g. Cohen's *d*, Pearson's *r*), indicating how they were calculated

Our web collection on statistics for biologists contains articles on many of the points above.

Software and code

Policy information about availability of computer code

Data collection	No software was used to collect the data.
Data analysis	R 3.6, R 4.0.3, CASAVA 1.8.2, BETSY, Trimmomatic 0.33, BWA 0.7.17-r1188, GATK 3.7, Picard 2.9.0, MuSE v1.0rc, SomaticSniper, Strelka, VarScan2, SnpEff, Annovar, ABSOLUTE, FACETS, Sequenza, DeconstructSigs, SigMA, STAR, HTSeq-count, fGSEA, DESeq2, GATHER, GraphPad PRISM 8

For manuscripts utilizing custom algorithms or software that are central to the research but not yet described in published literature, software must be made available to editors and reviewers. We strongly encourage code deposition in a community repository (e.g. GitHub). See the Nature Portfolio guidelines for submitting code & software for further information.

Data

Policy information about availability of data

All manuscripts must include a data availability statement. This statement should provide the following information, where applicable:

- Accession codes, unique identifiers, or web links for publicly available datasets
- A description of any restrictions on data availability
- For clinical datasets or third party data, please ensure that the statement adheres to our policy

RNA-seq data described in this study are deposited in the Gene Expression Omnibus database under accession GSE160568. The raw RNA-Seq and whole exome sequencing data are available under controlled access from the European Genome-phenome Archive under accession EGAD0000100827.

Field-specific reporting

Please select the one below that is the best fit for your research. If you are not sure, read the appropriate sections before making your selection.

☒ Life sciences ☐ Behavioural & social sciences ☐ Ecological, evolutionary & environmental sciences

For a reference copy of the document with all sections, see nature.com/documents/nr-reporting-summary-flat.pdf

Life sciences study design

All studies must disclose on these points even when the disclosure is negative.

Sample size	Samples were collected from all patients that participated in a previously published clinical trial.
Data exclusions	Five samples were excluded due to poor profiling of cancer tissue, as described in the manuscript.
Replication	N/A
Randomization	N/A. All patients received the same treatment, and this study was designed to identify correlates of response.
Blinding	N/A.

Reporting for specific materials, systems and methods

We require information from authors about some types of materials, experimental systems and methods used in many studies. Here, indicate whether each material, system or method listed is relevant to your study. If you are not sure if a list item applies to your research, read the appropriate section before selecting a response.

Materials & experimental systems

n/a	Involved in the study	
☒	☐	Antibodies
☒	☐	Eukaryotic cell lines
☒	☐	Palaeontology and archaeology
☒	☐	Animals and other organisms
☐	☒	Human research participants
☒	☐	Clinical data
☒	☐	Dual use research of concern

Methods

n/a	Involved in the study	
☒	☐	ChIP-seq
☒	☐	Flow cytometry
☒	☐	MRI-based neuroimaging

Human research participants

Policy information about studies involving human research participants

Population characteristics	We investigated tumors from breast cancer patients with germline mutations in BRCA1 or BRCA2 and were treated with single agent talazoparib as neoadjuvant therapy.
Recruitment	The patients had participated in a clinical trial that was previously described in a separate study. We used data from all patients in the trial.
Ethics oversight	This study was conducted under an IRB approved by the University of Texas MD Anderson Cancer Center.

Note that full information on the approval of the study protocol must also be provided in the manuscript.
